# Artificial Intelligence Applications in MRI for the Diagnosis and Management of Osteonecrosis of the Femoral Head: A Comprehensive Review

**DOI:** 10.3390/bioengineering13080942

**Published:** 2026-08-20

**Authors:** Federica Denami, Antonio Ammendolia, Alessandro de Sire, Nicola Marotta, Giorgia Lucia Benedetto, Elvira Immacolata Parrotta, Giovanni Cuda, Giorgio Gasparini, Michele Mercurio

**Affiliations:** 1 Department of Orthopaedic and Trauma Surgery, Magna Græcia University, “Renato Dulbecco” University Hospital, V.le Europa, 88100 Catanzaro, Italy; gasparini@unicz.it (G.G.); michele.mercurio@unicz.it (M.M.); 2 Research Center on Musculoskeletal Health, MusculoSkeletal Health@UMG, Magna Graecia University, 88100 Catanzaro, Italy; ammendolia@unicz.it (A.A.); alessandro.desire@unicz.it (A.d.S.); nicola.marotta@unicz.it (N.M.); 3 Physical and Rehabilitative Medicine, Department of Medical and Surgical Sciences, Magna Græcia University, 88100 Catanzaro, Italy; 4 Physical Medicine and Rehabilitation, Department of Experimental and Clinical Medicine, Magna Græcia University, 88100 Catanzaro, Italy; 5 Department of Medical and Surgical Sciences, Magna Græcia University, 88100 Catanzaro, Italy; giorgialucia.benedetto@studenti.unicz.it (G.L.B.); parrotta@unicz.it (E.I.P.); 6 Department of Experimental and Clinical Medicine, Magna Græcia University, 88100 Catanzaro, Italy; cuda@unicz.it

**Keywords:** deep learning, artificial intelligence, machine learning, ONFH, AVN, osteonecrosis, avascular necrosis, MRI, diagnosis

## Abstract

Osteonecrosis of the femoral head (ONFH) is a progressive and potentially disabling condition caused by compromised blood supply to the femoral head, leading to bone necrosis and collapse. Early diagnosis is essential to enable joint-preserving interventions and improve patient outcomes. Magnetic resonance imaging (MRI) is currently considered the most sensitive modality for early detection, whereas computed tomography (CT) provides superior assessment of subchondral bone integrity and structural collapse. In recent years, artificial intelligence (AI), particularly machine learning (ML) and deep learning (DL) techniques, has emerged as a promising tool to enhance diagnostic accuracy, automate lesion segmentation, and predict disease progression. This review aims to provide an overview of current AI applications in MRI for ONFH, focusing on early diagnostic, disease staging and classification, volumetric assessment, differential diagnosis and prognostic prediction. A total of 61 articles were initially identified, of which 13 studies (2021–2025) met the inclusion criteria. Results indicate that DL models, particularly convolutional neural networks (CNNs), achieve excellent diagnostic performance, with reported accuracies up to 98.4% and area under the curve (AUC) values reaching 0.98 for early-stage detection. Several models demonstrated performance comparable to or exceeding that of experienced clinicians, particularly in differentiating ONFH from other hip pathologies and in early disease recognition. AI algorithms also showed high accuracy in staging and classification (AUC up to 99.7% in internal validation), as well as in automated segmentation and volumetric assessment (Dice coefficients up to 0.89), enabling objective quantification of necrotic lesions. Furthermore, prognostic models integrating radiomics and ML techniques demonstrated promising results in predicting femoral head collapse (AUC up to 0.85). From a clinical perspective, AI appears to function primarily as a supportive tool, improving diagnostic consistency, efficiency, and reproducibility, and acting as a “second reader” capable of reducing variability among less experienced clinicians. However, significant limitations remain, including dataset heterogeneity, predominance of retrospective and monocentric studies, and limited integration of clinical data. In conclusion, AI-based MRI analysis shows strong potential to enhance the diagnosis, staging, and management of ONFH. Future research should focus on multicenter prospective validation, integration of multimodal clinical data, and development of explainable and generalizable models to facilitate widespread clinical adoption.

## 1. Introduction

Osteonecrosis of the femoral head (ONFH), also referred to as avascular necrosis, is a multifactorial disease characterized by impaired vascular supply to the femoral head, resulting in ischemia, bone necrosis, and progressive structural failure [1]. The condition predominantly affects young and middle-aged adults and is associated with significant morbidity, frequently leading to total hip arthroplasty if not diagnosed and managed at an early stage [2]. The pathophysiology of ONFH involves a complex interplay of vascular compromise, increased intraosseous pressure, and bone cell death [3,4]. Common etiological factors include prolonged corticosteroid use, excessive alcohol consumption, trauma, and systemic conditions such as coagulopathies and autoimmune diseases [5,6]. The disease typically progresses through a continuum of stages, from asymptomatic bone marrow alterations to subchondral fracture and femoral head collapse [7,8].

Accurate staging is essential for prognosis and treatment planning. Among the most widely used classification system is the Ficat and Arlet classification [9], based primarily on radiographic findings and clinical symptoms. More advanced imaging-based system, such as the Association Research Circulation Osseous (ARCO) classification [10], incorporate findings from radiography, MRI, and CT and provides more detailed stratification of disease severity, including lesion size and location. In particular, the extent of necrotic involvement of the weight-bearing surface has been shown to correlate strongly with the risk of femoral head collapse. MRI-based classifications further refine staging by identifying early marrow abnormalities, such as the characteristic “double-line sign” on T2-weighted images, which reflects the interface between necrotic and viable bone [11].

Imaging plays a central role in the diagnosis and management of ONFH. MRI is considered the gold standard for early detection due to its high sensitivity and ability to identify pre-radiographic changes [12,13,14]. CT, in contrast, provides superior spatial resolution for evaluating cortical bone and is particularly useful in detecting subchondral fractures and assessing the degree of collapse [15,16]. Despite these advances, interpretation of imaging findings remains challenging, especially in early-stage disease, and is subject to inter-observer variability.

In this context, artificial intelligence (AI) has emerged as a transformative approach in medical imaging. Deep learning (DL) models, particularly convolutional neural networks (CNNs), have demonstrated excellent performance in image classification, segmentation, and pattern recognition [17,18,19]. Their application to musculoskeletal imaging, including ONFH, offers the potential to improve diagnostic accuracy, standardize disease staging, and support prognostic assessment [20,21].

While generic reviews on the applications of AI in musculoskeletal imaging or broad orthopedic fields are increasingly available, they often provide generalized overviews without delving into the specific diagnostic and prognostic nuances of progressive bone ischemic conditions. To properly contextualize the novelty of this study, it is essential to examine the landscape of existing literature reviews. Currently, published papers predominantly fall into two categories. The first includes multi-modal systematic reviews evaluating deep learning applications across highly heterogeneous datasets, combining standard radiographs, CT, and MRI over vast historical timelines spanning several decades [22]. The second category [23] comprises clinical or epidemiological reviews that focus primarily on differential diagnosis and the clinical presentation of avascular necrosis, frequently discussing artificial intelligence only as a prospective, qualitative auxiliary tool rather than a quantitative diagnostic matrix. Nevertheless, an updated MRI-focused synthesis integrating the different stages of the ONFH clinical pathway remains clinically relevant. To our knowledge, the present review provides an updated MRI-focused synthesis of AI applications in ONFH, examining diagnostic and differential diagnostic performance, disease staging and classification, automated lesion segmentation and volumetric assessment, and prognostic prediction. By organizing the evidence according to these clinically distinct tasks, this review aims to clarify the potential role and current limitations of AI-based MRI analysis in supporting clinical decision-making in ONFH.

## 2. Materials and Methods

**Figure 1:**
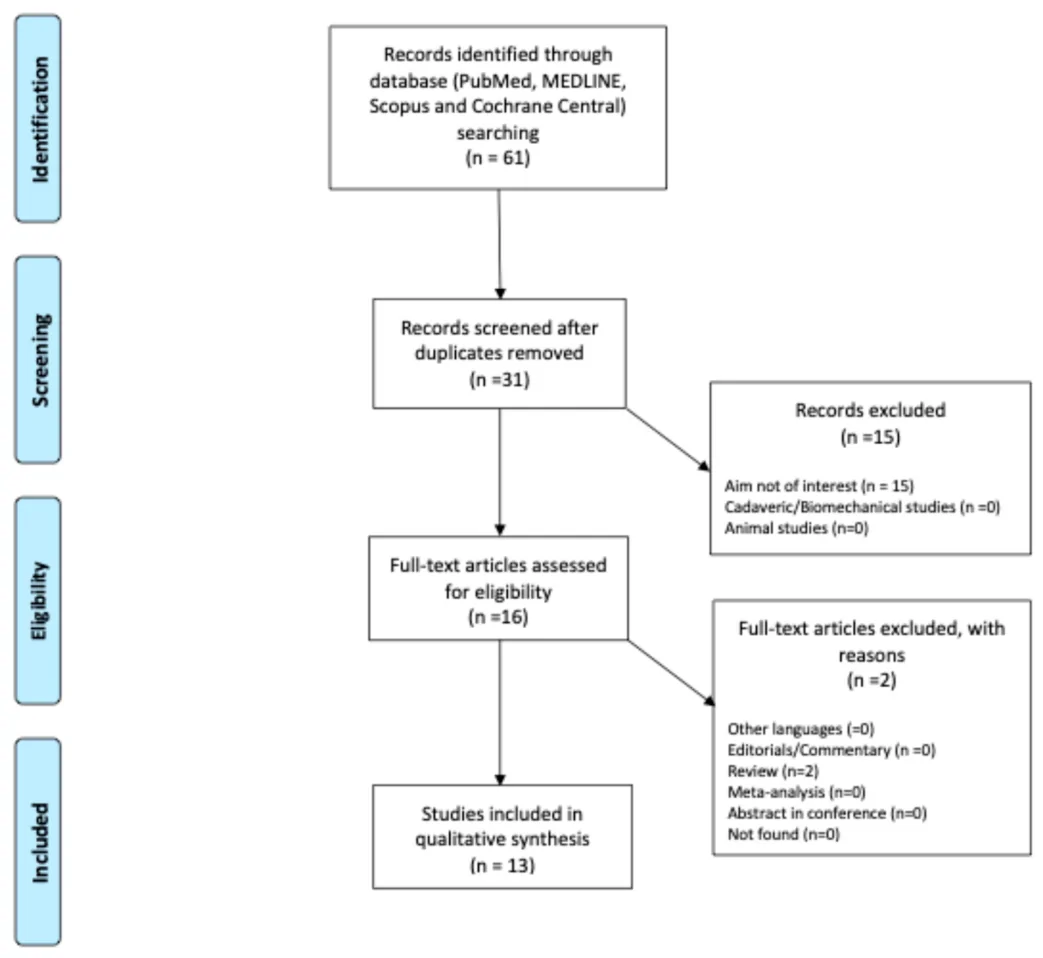
PRISMA flowchart illustrating the study selection process, including identification, screening, eligibility assessment, and final inclusion of studies in the systematic review.

A literature review was conducted and reported in accordance with the Preferred Reporting Items for Systematic Reviews and Meta-Analyses statement (PRISMA) [24] guidelines (Figure 1). A comprehensive literature search was performed in January 2026 using PubMed, MedLine, Scopus, and the Cochrane Central Register of Controlled Trials. The search strategy included the following terms: (“osteonecrosis of the femoral head” OR “ONFH” OR “avascular necrosis” OR “femoral head necrosis”) AND (“artificial intelligence” OR “machine learning” OR “deep learning” OR “convolutional neural network” OR CNN) AND (“magnetic resonance imaging” OR “MRI”). The final search strategy was independently reviewed by a second author before execution. The complete reproducible search strategies, together with the date of the final search, are provided in Supplementary Table S1.

Studies were included if they met the following criteria: (1) original research articles, observational studies, or clinical trials; (2) focused on AI applications for ONFH; (3) involving human subjects; (4) reporting outcomes related to diagnosis, segmentation, or prediction; (5) published in English within the last five years. Exclusion criteria comprised reviews, editorials, conference abstracts without full text, and preclinical studies not involving human participants. Studies including fewer than ten patients were excluded to ensure meaningful analysis. Only articles published in the last five years were considered. The systematic evidence synthesis was restricted a priori to studies published between January 2021 and December 2025. This five-year window was selected to provide a focused assessment of contemporary AI applications in MRI for ONFH, reflecting recent developments in model architecture, validation strategies, imaging pipelines, and clinically oriented AI applications. This temporal restriction was intended to define the scope of the review rather than to imply that earlier studies lack methodological or clinical relevance. Earlier literature was considered for contextual and historical purposes where appropriate but was not included in the systematic evidence synthesis. Two authors (FD and EP) independently screened records and abstracts to identify articles for inclusion, contacting a third senior author (MM) in cases of disagreement [25]. The reference list of each included article, as well as the available gray literature at our institution, were screened for potential additional articles. Other reviews, editorials, letters to the editor, and expert opinions were also considered but not included. Emphasis was placed on studies applying AI to diagnosis, imaging, outcome prediction, or treatment planning for ONFH. The included articles are summarized in Table 1.

Performance metrics were extracted and synthesized separately according to the clinical task. Diagnostic and classification studies were evaluated using task-appropriate discrimination and classification metrics (e.g., AUC/AUROC, accuracy, sensitivity, specificity, precision, and F1-score), segmentation studies using spatial overlap and boundary-based metrics (e.g., Dice coefficient, IoU, Hausdorff distance, and ASSD), and prognostic studies using prognostic discrimination, calibration, and clinical utility measures, when reported. Metrics derived from different tasks were not considered directly comparable (Table 2).

IEEE Xplore was initially considered; however, a pilot search yielded purely technical engineering frameworks lacking peer-reviewed human clinical validation data for ONFH, which fell outside the explicit medical scope of this review.

Risk of bias was independently assessed by three reviewers (FD, EP, and MM) using PROBAST + AI, the updated Prediction Model Risk of Bias Assessment Tool for studies using regression or artificial intelligence methods [39]. Given that the present review primarily focused on the reliability of reported model performance, the model-evaluation component of PROBAST + AI was applied. Four domains were assessed: participants and data sources, predictors, outcome, and analysis. Each domain was rated as low, high, or unclear risk of bias, and an overall judgment was derived from the domain-level assessments without calculating an aggregate numerical score. Particular attention was paid to patient-level data partitioning, potential pseudo-replication, the timing of data augmentation, the independence of validation datasets, reference-standard definition, and the appropriateness of task-specific performance measures. For segmentation studies, the outcome domain was assessed with reference to the ground-truth segmentation or annotation procedure. Disagreements between reviewers were resolved by consensus.

## 3. Results

**Figure 2:**
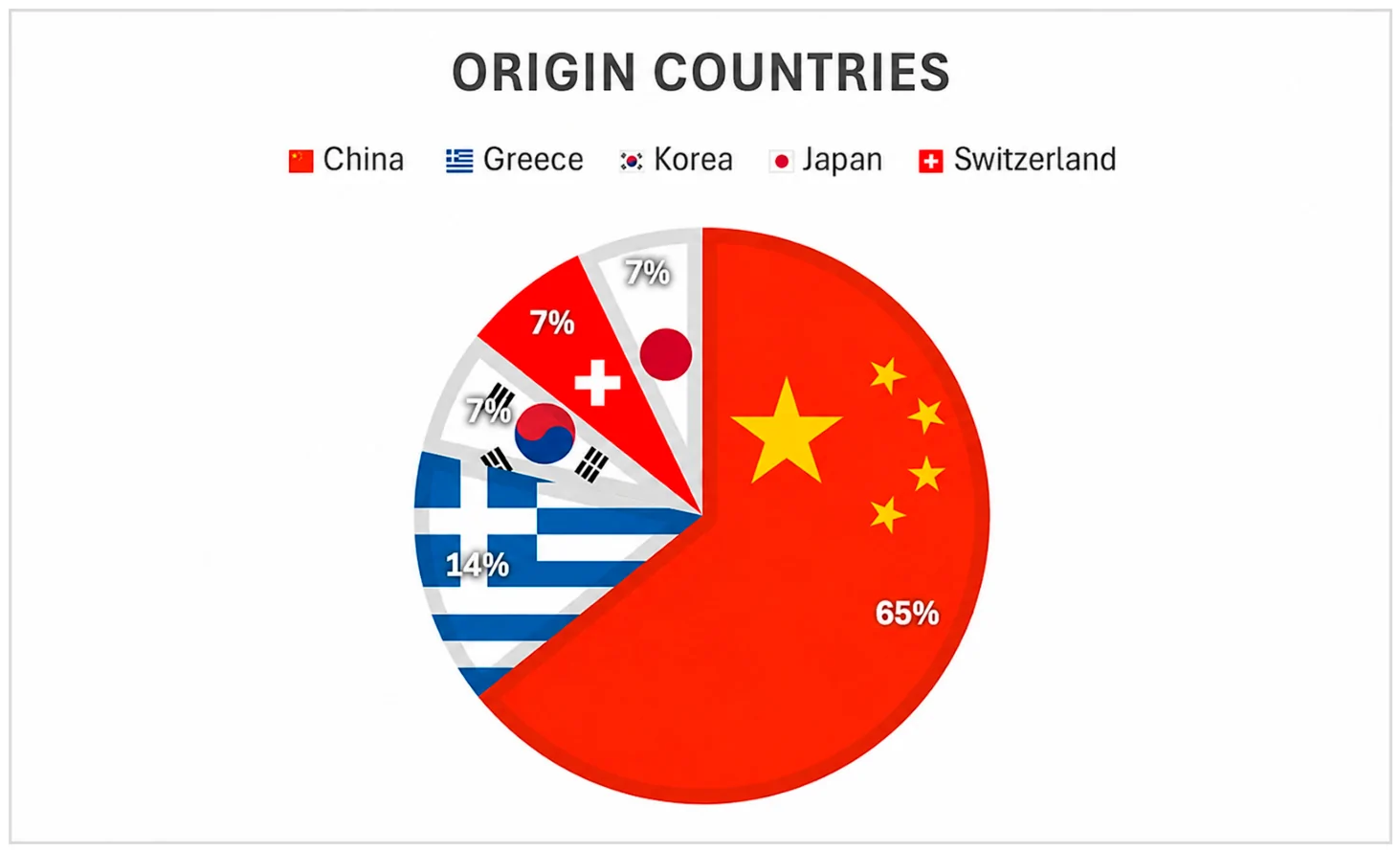
Geographic distribution of the included studies.

A total of 61 articles were identified through the initial search, resulting in 13 studies ([26,27,28,29,30,31,32,33,34,35,36,37,38]) that were included in the review (Figure 1). The selected articles originated from 5 countries, with China contributing the largest number (n = 9), followed by Greece (n = 2), and Korea, Japan and Switzerland with 1 each (Figure 2). The literature search highlighted the prominent role of specific research groups, most notably those led by Shen and Klontzas, who contributed three [33,34,35] and two studies [29,30] respectively, accounting for much of the high-quality evidence identified in this review.

**Figure 3:**
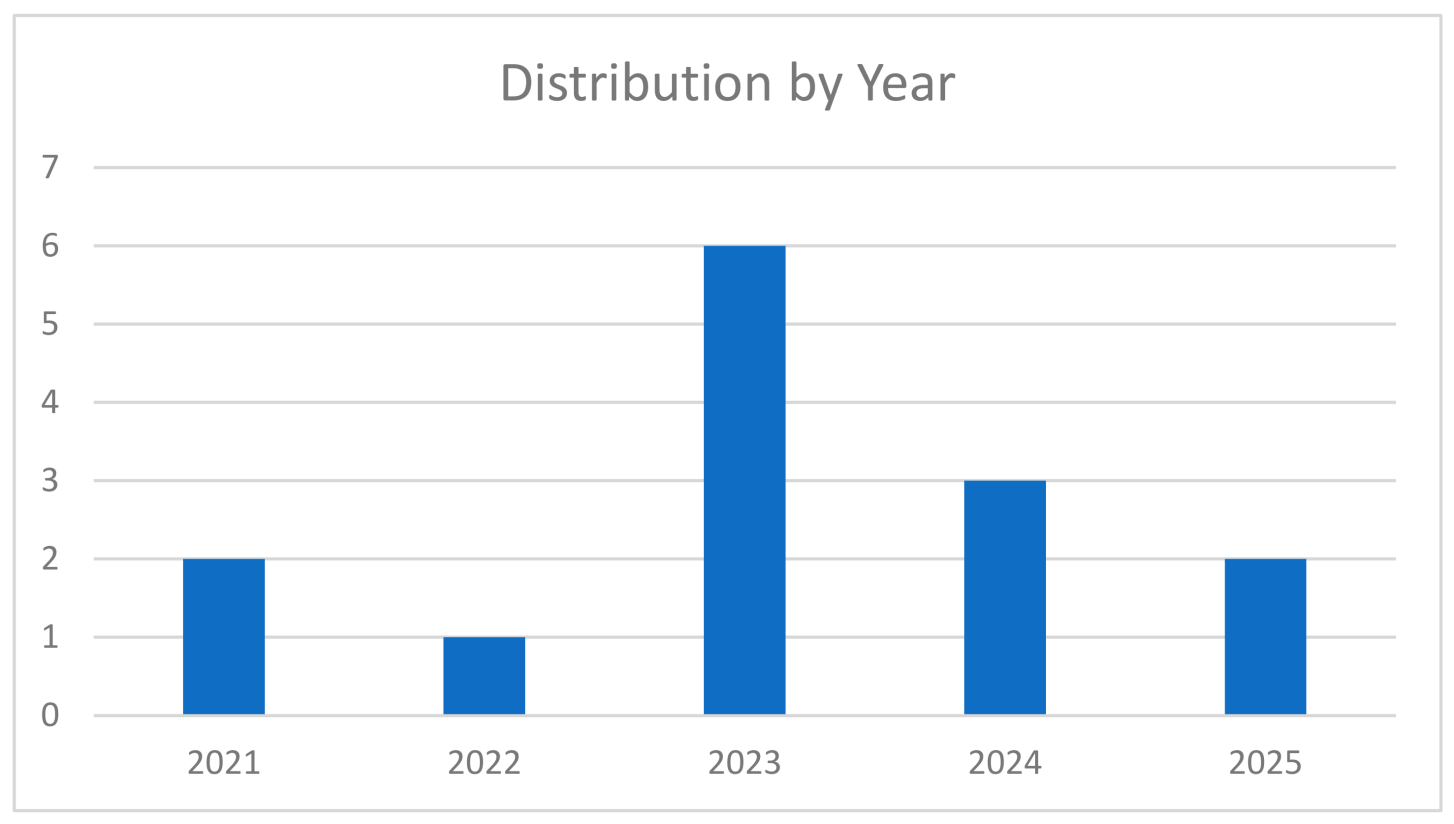
Distribution of included studies by publication year.

The review includes studies published between 2021 and 2025, showing a significant peak in research activity during 2023, with six studies published in that year alone (Figure 3). Figure 3 demonstrates a clear increase in the number of publications during the last five years, confirming the rapidly expanding interest in AI applications for MRI evaluation of ONFH. This trend reflects the increasing maturity of DL methodologies and highlights the emergence of AI as a relevant research topic within musculoskeletal imaging.

The number of patients enrolled across the included studies varied considerably, ranging from 26 to 794. Patient counts were considered separately from the number of MRI examinations, images, slices, or segments used for model development and evaluation, as multiple imaging observations may originate from the same patient and therefore do not represent independent subjects. These quantities are reported separately in Table 1. Regarding diagnostic performance, the DL model developed by Shen et al. [33] achieved the highest overall accuracy, while the model proposed by Klontzas et al. [30] demonstrated the superior discriminatory power with the highest AUC.

Independent external validation was reported only in a subset of the included studies, whereas several models were evaluated exclusively using internal test sets or data derived from a single institution. The characteristics and limitations of external validation are summarized for each study in Table 2. The strategies used to prevent data leakage and pseudo-replication varied across the included studies. Some studies explicitly implemented patient-level partitioning, ensuring that correlated observations from the same individual remained within the same dataset, whereas in others the level of data splitting was not clearly reported. This issue was particularly relevant in studies using multiple hips, slices, or MRI segments from individual patients. When patient-level separation could not be established from the original report, this was classified as unclear rather than assuming independence of imaging observations.

PROBAST + AI identified heterogeneous risk-of-bias profiles across the included studies (Table 3). One study was judged to have an overall low risk of bias, five an unclear risk, and seven a high risk. The analysis domain represented the most frequent source of concern, particularly because of small evaluation samples, insufficient reporting of patient-level data partitioning, potential pseudo-replication, and limited independent external validation. These findings indicate that the high performance reported by several models should be interpreted in the context of substantial methodological heterogeneity.

### 3.1. Diagnosis

The reviewed literature demonstrates a progressive transition from single-center model development toward multicenter validation and evaluation in more clinically heterogeneous diagnostic settings. A major trend involves benchmarking convolutional architectures directly against clinical experts to justify automated screening utility. Specifically, Shen et al. [33] established a large-scale benchmark using a retrospective dataset of 11,061 MR images, achieving a high diagnostic ceiling with an AUC of 0.98 and an accuracy of 98.4%. Critically, human–machine comparative trials revealed that while the system significantly outperformed resident and attending surgeons, its diagnostic metrics were statistical equivalents to senior orthopedic specialists. This comparative parity implies that while DL cannot yet replace high-level clinical intuition, its primary value lies in community screening and reducing diagnostic errors for less experienced clinicians. To move closer to realistic clinical environments, recent frameworks have transitioned toward multi-centre Deep Convolutional Neural Network (DCNN) systems trained on large-scale datasets across distinct institutions. Shen et al. [35] addressed this by developing a DCNN trained on 11,730 MRI segments derived from patients recruited across four institutions, explicitly introducing confounding hip pathologies—such as fractures, dislocations, and bone marrow edema—which typically distort standard convolutional layers. Despite the increased diagnostic difficulty, the model retained robust generalizability, yielding an internal AUC of 0.97 and a stable external validation AUC of 0.95, while demonstrating performance comparable to senior deputy chief physicians. Furthermore, Grad-CAM provided post-hoc visualization of image regions contributing to model predictions, with highlighted areas corresponding to necrotic regions, thereby improving visual interpretability. However, such saliency maps should not be considered evidence of causal or clinically valid reasoning and do not, by themselves, establish model reliability, robustness, or the absence of shortcut learning. However, a comparative technical analysis reveals substantial variations in model robustness based on network selection and data complexity. When evaluating standard architectures, Liu et al. [38] reported that ResNet18 achieved higher performance than the alternative architectures evaluated in their dataset (AlexNet and VGG16), with a total MRI detection rate of 99.44%. This finding should be interpreted as study-specific and does not establish the general superiority of ResNet18 over other architectures. For instance, the single-center study by Wang et al. [37] optimized a CNN on 3640 MRI segments, reaching an AUC of 0.97. However, a critical limitation of this framework is its strict restriction to distinguishing only healthy controls from verified ONFH cases, failing to simulate clinical scenarios where radiologists must differentiate early osteonecrosis from overlapping joint diseases. To overcome the pitfalls of binary isolation, contemporary strategies have successfully shifted toward CNN ensemble methodologies tailored for challenging differential diagnoses. Klontzas et al. [29] highlighted this utility by deploying a transfer-learning ensemble network (combining VGG-16, InceptionResNetV2, and InceptionV3) specifically to untangle the complex clinical boundary between TOH and early-stage ONFH. Evaluating an augmented dataset of 420 hips, the multi-architecture ensemble achieved an AUC of 0.9762 with a perfect precision of 100% for ONFH classification. Within this specific dataset, the multi-architecture ensemble achieved an AUC of 0.9762 with a precision of 100% for ONFH classification. These findings suggest that ensemble approaches may be useful in challenging differential-diagnosis settings; however, they do not establish the general superiority of multi-architecture ensembles over individual convolutional architectures. Similarly, Alkhatatbeh et al. [26] developed an interpretable radiomics-based ML model to differentiate between ONFH and hip osteoarthritis. The combined DL-based Radiomics (DLR) model using a single T2-weighted MRI sequence achieved superior AUC of 0.968 (95% CI: 0.909–1.000), with sensitivity of 95% and specificity of 92%, outperforming both the pure radiomics model (AUC 0.944) and the deep learning model (AUC 0.930). Decision curve analysis confirmed that the DLR model offered the greatest net clinical benefit in detecting early ONFH.

### 3.2. Disease Staging and Classification

Beyond binary detection, the automation of multi-class classification staging systems represents a critical technical pivot, though it exposes clear boundary conflicts across the literature. Klontzas et al. [30] advanced the field by testing a CNN ensemble (VGG-16, InceptionResNetV2, InceptionV3) across multiple sequences (T1-w, STIR, PD/T2 fs) to differentiate early (ARCO stages I–II) from advanced (ARCO stages III–IV) osteonecrosis. The architecture achieved an outstanding diagnostic ceiling with an AUC of 0.997, demonstrating performance comparable to expert radiologists even on external validation sets. This success highlights that ensemble methods successfully identify structural disruptions like the subchondral crescent sign. However, a persistent limitation in this domain is the reliance on single-view inputs, which fails to account for the frequently bilateral nature of early ischemic necrosis. To address this spatial limitation, contemporary staging frameworks have transitioned toward multi-scale geometric feature extraction and multi-view configurations. Li et al. [31] designed a MsgeCNN using axial T1-w MRI slices to automate both segmentation and necrotic ratio grading. While the model achieved a high segmentation accuracy of 97.73%, its staging classification accuracy dropped to 90.80%. The comparative analysis reveals that performance drops significantly near the structural thresholds separating early stages, particularly when transitioning from pre-collapse to early subchondral fracture. This finding underscores that while geometric embedding improves boundary definitions, standalone convolutional models still experience higher inter-model variability when staging subtle bone architectural changes compared to advanced multi-view systems. Further architectural scaling has led to the integration of deep convolutional frameworks tailored to specific regional staging guidelines, such as the Japanese Investigation Committee (JIC) matrix. Shen et al. [35] implemented a DenseNet framework trained on a large midcoronal T1WI/T2WI MRI dataset (842 patients) specifically for JIC classification and automated lesion localization. The model demonstrated robust internal accuracy (87.8%, AUC 0.90) and acceptable external generalizability (83.8%, AUC 0.87). However, as the model was developed according to the JIC classification system, its applicability to settings in which other staging systems, such as ARCO or Steinberg, are predominantly used remains uncertain and requires further validation.

### 3.3. Volumetric Assessment and Segmentation

Automated semantic segmentation represents the baseline requirement for turning qualitative radiological assessments into objective, operator-independent measurements. The included studies increasingly employed self-configuring and advanced 3D U-Net variants, which showed promising segmentation performance within the respective study settings. Ruckli et al. [32] evaluated the state-of-the-art nnU-Net framework on a combined protocol of coronal 2D PD-w and 3D T1 VIBE MRI sequences, achieving an average segmentation accuracy of 75 ± 15%. Crucially, the study showed strong spatial agreement with manual expert tracing, showing the potential of automated volumetric quantification to support objective lesion assessment relevant to joint-preserving treatment planning. However, the small sample size (26 patients) and reliance on a single MRI vendor may limit the generalizability of these findings. These factors could potentially contribute to variability in model performance, although their specific impact was not directly evaluated in the study. To mitigate sample constraints and improve boundary delineations near complex subchondral bone interfaces, recent studies have introduced optimized structural learning strategies. Uemura et al. [36] utilized a 3D Dynamic U-Net on coronal plane MRI (56 patients), achieving an automated staging and volumetric segmentation accuracy of 93.7%. While the dynamic feature scaling allowed the network to adapt to varying lesion sizes, the study remained constrained by a high risk of single-surgeon annotation bias.

### 3.4. Prognostic Prediction and Collapse Prediction

The final thematic cluster in the reviewed results represents the transition from static diagnostic triage to long-term prognostic forecasting, specifically predicting whether a lesion will progress to subchondral fracture and mechanical femoral head collapse. Methodologically, these prognostic frameworks yield the highest clinical utility when they pair automated volumetric segmentation with tabular clinical metrics, rather than relying strictly on isolated image features. Gao et al. [27] addressed this by establishing a pipeline that combines nnU-Net automated segmentation with a multi-model machine learning classifier (comparing LR, SVM, RF, KNN, XGBoost, and LightGBM) on T1-w MRI radiomics data. Among the pipelines tested, the LightGBM classifier achieved superior prognostic power with an AUC of 0.924 and a predictive accuracy of 76.5%. However, this study-specific finding does not establish a general superiority of tree-based gradient boosting methods over linear models for high-dimensional radiomic data. Moreover, because the study relied on a retrospective design, the model remains insulated from real-world imaging artifacts that routinely occur during prospective clinical follow-ups.

The necessity of defining strict temporal boundaries is further illustrated when comparing hybrid imaging frameworks. Kim et al. [28] deployed a VGG11 U-Net featuring an attention fusion network trained on multi-protocol 3D MRI (T1, STIR) to achieve MR-only staging and prediction, yielding an AUC of 0.92 on a cohort of 45 patients. By leveraging self-supervised learning, the architecture successfully extracted deep structural features from small samples. Conversely, when these advanced frameworks are compared to structurally isolated models, clear clinical limitations emerge. For instance, the multi-modal study by Yan Liu et al. [38] compared eight computational methods, demonstrating that a ResNet18 architecture combining T1WI MRI and CT data achieved a peak total detection rate of 98.23%. However, the relatively short follow-up period limits the ability to assess longer-term treatment efficacy and structural outcomes. For prognostic models aimed at predicting femoral head collapse, longer follow-up periods may provide a more clinically meaningful assessment of disease progression. In the present review, a 24-month follow-up was considered a reasonable reference timeframe; however, the appropriate follow-up duration should be determined according to the specific outcome, disease stage, intervention, censoring strategy, and clinical objective.

## 4. Discussion

The findings of this review indicate that AI represents a promising adjunctive tool in the diagnosis and classification of ONFH, particularly in the context of imaging-based evaluation.

External validation represents a critical issue across the included studies. Although some models were evaluated using independent external datasets, external validation remains limited across the overall evidence base, with several studies relying primarily on internal validation or single-center datasets. Consequently, high performance observed during internal testing may not necessarily translate to different institutions or imaging environments. The available external validation results are encouraging, but they should be interpreted cautiously given the limited number and diversity of independent validation cohorts. Further validation across geographically and institutionally independent populations, different MRI vendors and field strengths, and heterogeneous acquisition protocols is required to assess model robustness and generalizability and to quantify the impact of domain shift. An additional methodological concern is the potential for data leakage and pseudo-replication. MRI datasets may contain multiple slices, sequences, or bilateral hips derived from the same patient, and these imaging units cannot be considered statistically independent. If correlated observations from the same individual are distributed across training and test sets, model performance may be optimistically estimated because patient-specific information can be shared between datasets. In the included literature, some studies explicitly adopted patient-level splitting or independent external validation, whereas others did not provide sufficient information to determine whether correlated imaging observations were kept within the same partition. Similarly, reporting of the timing of data augmentation relative to dataset splitting was inconsistent. Consequently, absence of reported patient-level partitioning should not be interpreted as evidence that leakage occurred, but it limits confidence that this source of bias was fully excluded. Future studies should explicitly perform and report patient-level partitioning before augmentation and ensure that external validation cohorts are independent at both patient and institutional levels.

An important observation is the marked geographical concentration of the available evidence, with the majority of included studies originating from China [40]. This may limit the generalizability of the reported models because potential sources of domain shift extend beyond geography itself and may include differences in population characteristics and disease etiology, MRI scanner manufacturer and field strength, acquisition and reconstruction protocols, institutional workflows, annotation practices, disease prevalence, and staging systems. Consequently, model performance observed in one geographic or institutional setting may not necessarily be reproduced in populations or imaging environments with different characteristics. Future studies should therefore prioritize geographically diverse, multicenter datasets and independent external validation across different scanners, protocols, and clinical settings [41]. A critical appraisal of the gathered evidence also reveals substantial methodological and clinical limitations that currently preclude widespread implementation of AI in ONFH management. Despite the outstanding performance metrics reported—such as CNN validation accuracies reaching 98.4%—the available evidence relies predominantly on retrospective datasets. Retrospective evaluation may yield optimistic estimates of clinical performance because of selection bias, controlled acquisition conditions, and limited representation of real-world distribution shifts. Most studies lack prospective, real-world validation, where variations in image quality, artifacts, patient characteristics, and atypical early-stage presentations may affect model performance.

Furthermore, a persistent technical bottleneck is the “black box” nature of these models. Although some authors integrated interpretability tools like Grad-CAM, there remains a distinct lack of integration between pixel-level MRI features and clinical/biochemical risk factors (e.g., cumulative corticosteroid dosages, metabolic status) [42]. However, these post-hoc visualization methods provide information on model attention rather than validated explainability and do not demonstrate causal reasoning or exclude reliance on spurious imaging features. Without multimodal data fusion, AI tools remain strictly secondary diagnostic readers rather than autonomous clinical decision support systems. Finally, the clinical utility of volumetric segmentation (e.g., dynamic U-Net frameworks yielding Dice coefficients of 0.89) is heavily constrained by the lack of standardized open-source codebases, which prevents independent multi-center benchmarking and perpetuates center-specific overfitting.

Recent reviews have provided important syntheses of AI and deep learning applications in osteonecrosis, including analyses encompassing multiple imaging modalities and studies primarily focused on diagnostic and classification performance [43,44]. The present review complements this literature by adopting a specifically MRI-focused and clinically oriented perspective, integrating evidence across early and differential diagnosis, disease staging and classification, lesion segmentation and volumetric assessment, and prognostic prediction. This approach provides an updated overview of how AI-based MRI analysis may support different stages of the clinical management of ONFH.

From a clinical perspective, although AI models demonstrate high levels of accuracy, their performance does not significantly exceed that of experienced clinicians [45]. Importantly, this finding should be interpreted considering that many AI systems are trained on datasets labeled by human experts. Under the specific experimental conditions of the included studies, AI models often demonstrated diagnostic performance comparable to that reported for radiologists or orthopedic surgeons. In this context, AI can be seen as a reflection and standardization of expert knowledge rather than a truly independent diagnostic entity [46]. At the same time, it is important to acknowledge one of the distinctive strengths of AI systems. As highlighted by Shen et al. [35], AI can recognize complex image patterns and mathematical motifs that are not perceptible to the human eye, thereby facilitating the detection of subtle and complex imaging features. This capability may be particularly relevant in the early stages of ONFH, where radiological signs can be minimal or difficult to interpret, and suggests a potential role for AI in supporting earlier diagnosis. However, our analysis highlights the role of DL as a clinical “equalizer” in the diagnosis of early-stage ONFH. Studies directly comparing AI performance with clinicians [34,37] reported that CNN-based models achieved diagnostic performance comparable to that of experienced musculoskeletal radiologists and, in some settings, higher than that of less experienced readers. This is particularly relevant in ARCO stages I and II, where necrotic changes are often subtle and easily overlooked. By acting as a high-performance “second opinion”, these findings suggest that AI-assisted interpretation may potentially reduce variability related to reader experience, particularly in non-specialized settings, although this potential benefit requires prospective validation in real-world clinical practice. However, these findings should be interpreted within the specific conditions of each reader study, including differences in reader expertise, case selection, and the imaging or clinical information available during assessment.

At present, therefore, AI should be considered primarily as a supportive tool [47]. Its principal advantages lie in improving efficiency and consistency. For instance, AI systems can significantly reduce processing times, as demonstrated by Shen et al. [35], who reported an average analysis time of approximately two seconds per case, and can minimize operator-dependent variability in image interpretation.

Across the included studies, a wide range of AI architectures was employed, reflecting both the rapid evolution of the field and the heterogeneity of the research approaches. Most studies relied on DL models, particularly CNNs, although traditional ML algorithms were also frequently used, especially for predictive tasks [48]. Among DL approaches, several CNN-based architectures were adopted for image classification and feature extraction [18]. Commonly used backbone networks included ResNet variants, particularly ResNet18, as well as DenseNet-based models, often referred to as deep convolutional neural networks (DCNNs). Other advanced architectures such as ResNeSt were also implemented, aiming to improve feature representation through enhanced attention mechanisms. In addition, multiple studies explored well-established CNN architectures including VGG-16, VGG11, InceptionV3, and InceptionResNetV2, either individually or in comparative frameworks to evaluate performance across different network designs.

Segmentation tasks were predominantly addressed using U-Net-based architectures [49]. Both the original U-Net and its more advanced variant, nnU-Net, were widely utilized due to their strong performance in medical image segmentation. In some cases, hybrid models such as VGG11 U-Net were developed to combine the feature extraction capabilities of classification networks with the localization strength of segmentation frameworks. The nnU-Net, in particular, was frequently applied for automated segmentation of the femoral head, demonstrating robust and reproducible results across datasets.

Beyond standard CNN architectures, some studies introduced more specialized models, such as MsgeCNN, designed to capture multi-scale or context-aware features, further highlighting the ongoing efforts to optimize performance for complex imaging patterns. In parallel, several studies employed traditional ML algorithms, particularly for prognostic tasks such as predicting femoral head collapse. These approaches typically relied on extracted imaging features or clinical variables as input. The most commonly used models included support vector machines (SVMs), random forests, logistic regression, k-nearest neighbors (KNN), Naive Bayes, and gradient boosting methods such as XGBoost and LightGBM. These ML models were often used either as standalone predictors or in combination with DL-derived features, reflecting a hybrid approach that leverages both representation learning and classical statistical methods [50].

Overall, the literature demonstrates a clear predominance of CNN-based architectures for diagnostic and classification purposes, while traditional ML models remain particularly relevant in predictive modeling [51]. This diversity of approaches underscores the absence of a standardized methodology and highlights the need for future studies to identify the most clinically effective and generalizable architectures.

The future of AI in ONFH appears to lie beyond simple morphological analysis, moving toward the integration of multi-modal data and radiomic features. The works of Gao et al. [27] and Kim et al. [28] illustrate that combining structural MRI data with texture analysis across multiple sequences (T1, T2, STIR) provides a more holistic view of the disease’s biological state. Radiomics, in particular, can identify sub-visual patterns of bone marrow heterogeneity that are predictive of femoral head collapse [52]. This augmented diagnostic approach suggests that the next generation of AI tools will not only show where the necrosis is but will also provide a personalized prognostic signature, estimating the risk of collapse with an accuracy that exceeds current clinical scores like the modified Kerboul angle.

To address the limitations associated with small dataset sizes, some authors adopted strategies such as data augmentation, transfer learning, and domain adaptation techniques. Transfer learning represents a critical methodological innovation that has enabled successful application of DL to medical imaging despite the typically limited availability of large annotated medical imaging datasets [53]. The classical transfer learning approach involves two stages: initial pretraining on a large, general-purpose dataset such as ImageNet, followed by fine-tuning on the target domain using the available medical imaging data. ImageNet, comprising over 14 million labeled images across thousands of object categories, provides an extraordinarily rich source of visual features that CNNs learn to extract. These learned features—including edge detection, texture discrimination, and shape recognition—exhibit remarkable transferability to medical imaging domains. The effectiveness of transfer learning in ONFH applications has been demonstrated through studies showing that ImageNet-pretrained networks, when properly fine-tuned, achieve excellent performance on external validation cohorts, suggesting genuine transfer of learned features from general-purpose to domain-specific image analysis.

A significant trend emerging from this review is the transition of ONFH assessment from traditional qualitative observation to high-precision automated quantification. While conventional MRI interpretation relies on the radiologist’s ability to identify specific signs, such as the double-line sign, recent AI advancements, particularly 3D U-Net [32,36], allow for the rapid volumetric measurement of necrotic lesions. This shift may be clinically relevant, as AI-driven volumetry provides objective quantitative information on the extent of femoral head involvement and may complement conventional staging. Such quantitative assessment could potentially support treatment planning by reducing some of the subjectivity associated with manual lesion estimation.

The methodological appraisal of the included evidence revealed heterogeneous risk-of-bias profiles, indicating that the reported performance of AI models should be interpreted with appropriate caution. PROBAST + AI identified the analysis domain as a frequent source of concern, particularly because of small evaluation samples, insufficient reporting of patient-level data partitioning, potential pseudo-replication, and limited independent external validation. Nevertheless, several studies incorporated methodological safeguards, including patient-level splitting, independent external validation, and consensus-based expert labeling, which strengthen confidence in specific aspects of model evaluation and reference-standard definition. These methodological considerations are particularly relevant given the technological evolution observed across the literature, from conventional 2D classification approaches toward 3D volumetric assessment and multimodal models. However, methodological rigor and high predictive performance do not, by themselves, address the interpretability limitations of AI models or constitute evidence of validated explainability. Moreover, the predominance of retrospective study designs, together with heterogeneous imaging protocols and validation strategies, continues to limit the generalizability of the available evidence. Future research should therefore prioritize prospective, multicenter studies with clearly defined patient-level data partitioning, standardized reporting, and independent external validation to determine whether the promising performance observed in current studies can be reliably translated into real-world clinical practice.

DL systems have demonstrated the potential to assist surgeons with less experience in ONFH diagnosis, as evidenced by studies from Shen and colleagues [33,34,35]. With performance comparable to senior surgeons and superior to residents, these systems could be particularly valuable in large-scale screening and in communities with a shortage of specialized experts. Studies with external validation, such as that by Klontzas et al. [30], have demonstrated that deep learning models can maintain high performance even when tested on data from different institutions and countries. These findings provide encouraging evidence regarding model generalizability, although broader independent validation remains necessary before widespread clinical implementation.

It is also worth noting that the study of ONFH extends beyond MRI alone. The literature includes investigations based on both histological and radiographic analyses, which have contributed to a deeper understanding of the pathophysiology and progression of the disease. While imaging remains central to clinical diagnosis and staging, histopathological studies provide important complementary insights.

### Limitations and Future Perspectives

Several limitations emerge from the current body of literature. Most studies are monocentric and retrospective in design, which limits the generalizability of their findings. In addition, many studies rely on relatively small datasets, often supplemented by data augmentation techniques to improve model performance. Expanding research across different geographic regions and healthcare systems will be essential to ensure external validity and to reduce potential biases related to ethnicity, imaging protocols, and disease prevalence.

A key limitation of the AI models themselves, particularly CNN-based approaches, is their reliance exclusively on imaging data, primarily MRI. These models do not incorporate essential clinical information such as patient history and physical examination findings, which are integral to real-world diagnostic reasoning. As a result, their decision-making process does not fully align with the comprehensive approach adopted by clinicians [54]. Future developments in the application of AI to ONFH are likely to focus on improving both the clinical relevance and generalizability of existing models. One of the most important directions will be the integration of multimodal data. Current AI systems rely predominantly on imaging, particularly MRI, but future models should incorporate clinical variables such as patient history, risk factors, laboratory findings, and physical examination data [55,56]. Such an approach would better reflect real-world diagnostic reasoning and could significantly enhance model performance and clinical applicability.

In addition, future AI systems may evolve from purely diagnostic tools to comprehensive decision-support platforms [57,58]. Beyond detecting and classifying ONFH, these models could assist in prognostic stratification, prediction of femoral head collapse, and selection of optimal treatment strategies, including joint-preserving procedures versus arthroplasty. The integration of longitudinal data may allow for dynamic monitoring of disease progression and treatment response. Technical advancements will also likely improve model transparency and interpretability. The development of explainable AI (XAI) techniques [59] could help clinicians better understand how algorithms reach their conclusions, thereby increasing trust and facilitating clinical adoption. This is particularly important in a field where decision-making has significant therapeutic implications. Finally, collaboration between clinicians, radiologists, data scientists, and regulatory bodies together with prospective real-world validation, will be crucial before the safe and effective implementation of AI in clinical practice [60,61]. Standardization of imaging protocols, annotation methods, and reporting outcomes will further contribute to the development of robust and reproducible models. Future efforts should aim to create more integrated, generalizable, and clinically meaningful AI systems capable of supporting the entire patient care pathway.

An additional limitation is the restriction of the systematic search to studies published between 2021 and 2025. Although this predefined temporal window was selected to focus the review on contemporary AI methodologies and their current clinical applications, it may have introduced temporal selection bias and excluded earlier foundational studies or relevant evidence on model development and validation. Therefore, the findings of this review should be interpreted as a synthesis of recent evidence rather than a comprehensive historical account of AI development in ONFH imaging.

The substantial heterogeneity of clinical tasks and reported performance metrics precluded direct quantitative comparison across models. In particular, classification, segmentation, and prognostic metrics measure different aspects of model performance and should not be interpreted interchangeably; therefore, comparisons were restricted to models addressing comparable tasks and outcomes.

## 5. Conclusions

Recent developments in deep learning and artificial intelligence have enhanced the approach to diagnosis, classification, and prognosis of osteonecrosis of the femoral head. From automated volumetric segmentation methods to attention networks for multimodal staging, from differential diagnosis to collapse prediction, the landscape of AI systems based on MRI has become sophisticated and clinically relevant.

The performance of these systems frequently matches that of expert radiologists, with the added advantage of providing assistance to less experienced clinicians in scenarios where experts are unavailable. Improved accessibility to MRI, together with the external validation reported in some studies, supports the potential clinical applicability of these systems. However, prospective multicenter validation in real-world clinical settings remains necessary before widespread clinical implementation. With these continued advances, AI systems represent an unprecedented opportunity to improve clinical outcomes in patients with ONFH, facilitating early diagnosis and optimal therapeutic decisions that could prevent progression to collapse and the need for surgery.

## Figures and Tables

**Table 1:** Included studies.

N	Authors (Year) [Ref] Country	AI Architecture	Primary Aim	N Patients	N Hips/MRI Units
1	Alkhatatbeh et al. (2025) China [26]	ML (SVM, RF, LR, KNN, XGBoost, Naive Bayes)	Construct and evaluate several Radiomics-based machine learning models using MRI to differentiate between OA and ONFH and compare their efficacies to those of medical experts.	140 (70 ONFH; 70 OA)	140 MRI scans
2	Gao et al. (2024) China [27]	nnU-Net for segmentations + ML algorithms for predicting femoral head collapse, (LR, SVM, RF, KNN, XGBoost, LightGBM)	Build an automatic femoral head collapse prediction pipeline for ONFH based on MRI radiomics	NR as unique patient count	Segmentation: 222 hips; prognosis: 206 hips
3	Kim et al. (2023) Republic of Korea [28]	VGG11 U-Net	Propose a DL model for MR-only ONFH staging, which can reduce additional cost and radiation exposure from the acquisition of CT images.	45 initially; 35 eligible after exclusions	69 femoral heads; 53 train/16 test
4	Klontzas et al. (2022) Greece [29]	CNNs (VGG-16, InceptionResNetV2, and InceptionV3)	Develop a DL methodology for the accurate differentiation between ONFH and TOH	174 (107 TOH; 67 ONFH)	213 hips (109 TOH; 104 ONFH)
5	Klontzas et al. (2023) Greece [30]	CNNs (VGG-16, InceptionResnetV2, InceptionV3)	Develop a DL methodology that distinguishes early from late stages of ONFH to determine treatment decisions.	Development cohort + independent external cohort	External test: 49 hips
6	Li et al. (2023) China [31]	MsgeCNN	Develop an automated system for segmenting the femoral head and necrotic regions from MRI images and grading ONFH based on the proportion of necrotic tissue	261	261 axial T1-w slices; one slice/patient
7	Ruckli et al. (2023) Switzerland [32]	nnU-Net	To automatically segment FHN based on routine hip MRI for improved surgical decision-making.	26	30 hips
8	Shen et al. (2023) China [33]	ResNeSt	Detect early ONFH and evaluate its feasibility in the clinic.	399 (172 ONFH; 227 normal)	11,061 MRI segments: 2279 ONFH + 8782 normal
9	Shen et al. (2023) China [34]	DCNN (DenseNet)	Develop a DCNN for detecting early ONFH from various hip pathologies	794	1588 hips; 11,730 MRI segments (5379 ONFH; 6351 non-ONFH)
10	Shen et al. (2024) China [35]	DenseNet	Perform the classification of ONFH through a deep learning approach using JIC classification system.	842	1337 hips; 1806 mid-coronal segments
11	Uemura et al. (2025) Japan [36]	3D Dynamic U-Net	Develop and validate a DL-based method for segmenting necrotic lesions using MRI; and to confirm the accuracy of the developed model in automatically classifying necrotic lesions according to the Steinberg classification.	56	63 hips
12	Wang et al. (2021) China [37]	U-Net	Detect early-stage ONFH	110	29 bilateral + 81 unilateral; 3640 MRI segments
13	Yan Liu et al. (2021) China [38]	ResNet18	Compare eight methods of detecting ONFH	360	192 unilateral + 168 bilateral patients; MRI examinations

Osteonecrosis of the femoral head (ONFH), transient osteoporosis of the hip (TOH), femoral head necrosis (FHN), Magnetic Resonance Imaging (MRI), convolutional neural network (CNN), Densely connected convolutional network (DCNN), Support Vector Machines (SVMs), visual geometry (VGG), multiscale geometric embedded convolutional neural network (MsgeCNN), K-Nearest Neighbors (KNN), eXtreme Gradient Boosting (XGBoost), Light Gradient Boosting Machine (LightGBM), Japanese Investigation Committee (JIC), Number (N), Random Forest (RF), Logistic Regression (LR).

**Table 2:** MRI characteristics, preprocessing procedures, validation strategies, ground-truth annotation, and task-specific performance metrics of the included studies.

N	Authors (Year) [Ref] Country	MRI Characteristics	Preprocessing/ Augmentation	Validation and Splitting Strategy	Ground Truth/ Annotation	Task-Specific Performance Metrics
1	Alkhatatbeh et al. (2025) China [26]	T2WI	Radiomic feature extraction and feature selection; augmentation NR	98 train/42 test; 10-fold CV during feature selection; no external validation	Senior MSK radiologist; clinical and MRI findings	Classification: test AUC ≈0.95–0.97; Sens 0.937; Spec 0.885; calibration and DCA reported
2	Gao et al. (2024) China [27]	Coronal T1-w TSE; 1.5/3 T; Siemens/GE	Radiomics preprocessing; ADASYN applied to training data for prognostic model	Segmentation: 190 train/32 test + 5-fold CV; prognosis: 155 hips from Center 1 train/51 hips from Center 2 external test	Manual ROI by senior surgeon; collapse determined during radiographic follow-up	Segmentation: Dice 0.848; HD95 3.794; ASD 0.554. Prognosis: external AUC 0.851; Sens 0.833; Spec 0.727; DCA reported
3	Kim et al. (2023) Republic of Korea [28]	Five MRI protocols; Siemens Skyra 3.0 T	Resampling, registration and ROI extraction; MR–CT self-supervised pretraining	Patient-wise hold-out + 5-fold CV; both femoral heads from the same patient kept in the same set	MSK radiologist assessment/consensus	Staging/classification: AUROC ≈ 0.89–0.92 depending on model/configuration
4	Klontzas et al. (2022) Greece [29]	Mid-coronal STIR; 1.5/3 T; multiscanner/multicenter	Grey-level harmonization, histogram normalization; rotation and horizontal flipping	70:30 training/validation; testing cohort used for validation; patient-level independence not clearly reported	Clinical + MRI reference standard with ≥1-year follow-up; MSK radiologist (35 years); consensus with referring surgeon	Classification: ensemble AUC 0.9762; sensitivity/specificity, precision, recall and F1 reported
5	Klontzas et al. (2023) Greece [30]	Mid-coronal STIR; additional MRI sequences acquired	Preprocessing and augmentation reported	Internal development/testing + independent external validation from another country	Two MSK radiologists (40 and 10 years), independent assessment + consensus	Classification: internal ensemble AUC 0.997; external AUC 0.855 (95% CI 0.722–0.939)
6	Li et al. (2023) China [31]	Axial T1-w MRI	Rotation and gamma correction applied to training samples	5-fold CV	Manual annotation by three physicians; reviewed by an experienced physician	Segmentation: Acc 97.73%; Sens 91.17%; Spec 99.40%; PPV 96.98%; Dice and HD reported. Classification: Acc 90.80%
7	Ruckli et al. (2023) Switzerland [32]	Coronal 2D PD-w TSE + 3D T1 VIBE; Siemens Skyra 3 T	nnU-Net standardized preprocessing	5-fold CV; single-center; patient-level grouping of multiple hips not clearly reported; no external validation	Manual expert segmentation	Segmentation: necrosis Dice 0.75 ± 0.15; unaffected-bone Dice 0.91 ± 0.05; volumetric agreement reported
8	Shen et al. (2023) China [33]	Coronal MRI	Preprocessing reported	Segments divided 70:20:10 into train/validation/test; patient-level separation not explicitly reported; no external validation	Diagnosis based on imaging/expert assessment	Classification: AUC 0.98; Acc 98.4%; Sens 97.6%; Spec 98.6%
9	Shen et al. (2023) China [34]	Multicenter coronal MRI	Preprocessing/augmentation reported	Multicenter development with internal and external test datasets; patient/hip/segment-level independence incompletely reported	Expert clinical/imaging reference standard	Classification: internal AUC 0.97 (95% CI 0.93–1.00), Acc 96.6%; external AUC 0.95 (95% CI 0.91–0.99), Acc 95.2%; precision, recall and F1 reported
10	Shen et al. (2024) China [35]	T1WI/T2WI; 1.5/3 T; UIH, Siemens, Philips, GE	Standardized preprocessing	1030 train/148 validation/294 internal test/334 external test; external cohort from separate institution	Two advanced orthopaedic surgeons; consensus	Classification: internal Acc 87.8%, macro-AUC 0.90; external Acc 83.8%, macro-AUC 0.87
11	Uemura et al. (2025) Japan [36]	3D-SPGR; GE 1.5 T	3D image preprocessing	4-fold CV: 42 train/5 validation/16 test per fold; patient-level grouping of bilateral hips not clearly reported; no external validation	Manual segmentation by orthopaedic surgeon > 10 years; second surgeon > 15 years for interobserver analysis	Segmentation: necrosis Dice 0.89 (IQR 0.85–0.92); ASD 0.76 mm. Grading: Acc 93.7%; weighted κ 0.98
12	Wang et al. (2021) China [37]	MRI	Image preprocessing reported	Patient-level split: 77 patients/2556 segments training; 33 patients/1084 segments test	Manual contours by three orthopaedic surgeons; reviewed by five experts	Detection/classification: AUC 0.97; Sens 0.97; Spec 0.95
13	Yan Liu et al. (2021) China [38]	MRI	Preprocessing reported	Train/test strategy reported incompletely for determination of patient-level separation	Consensus evaluation by experienced imaging physicians	MRI classification: ResNet18 Acc 98.23%; detection rate 99.44%; sensitivity and specificity reported

Acc, accuracy; ASD, average surface distance; AUC, area under the curve; AUROC, area under the receiver operating characteristic curve; CI, confidence interval; CV, cross-validation; DCA, decision-curve analysis; HD, Hausdorff distance; HD95, 95th percentile Hausdorff distance; IQR, interquartile range; MRI, magnetic resonance imaging; MSK, musculoskeletal; NR, not reported; PPV, positive predictive value; ROI, region of interest; Sens, sensitivity; Spec, specificity; STIR, short tau inversion recovery; TSE, turbo spin echo.

**Table 3:** PROBAST + AI risk-of-bias assessment of the included studies. Risk-of-bias judgments were based on the PROBAST + AI model-evaluation framework. Overall judgments were derived from domain-level assessments rather than from a numerical summary score. “Unclear” indicates insufficient information in the original publication to confidently determine the risk of bias and should not be interpreted as evidence that bias occurred. Abbreviations: L, low risk of bias; U, unclear risk of bias; H, high risk of bias; RoB, risk of bias; PROBAST + AI, Prediction Model Risk of Bias Assessment Tool for Artificial Intelligence.

Study	Participants & Data Sources	Predictors	Outcome	Analysis	Overall RoB	Main Rationale for Overall Judgment
Alkhatatbeh et al. (2025) [26]	U	L	H	H	H	Retrospective dataset; limited validation and concerns regarding outcome/reference-standard assessment and analytical robustness
Gao et al. (2024) [27]	L	L	L	L	L	Independent center-based external testing for prognosis; appropriate task-specific evaluation and clearly separated development/test cohorts
Kim et al. (2023) [28]	U	L	L	H	H	Explicit patient-wise splitting minimizes leakage, but the small development and test samples substantially limit analytical robustness
Klontzas et al. (2022) [29]	U	L	L	H	H	Multicenter/multiscanner data, but limited independent validation and uncertainty regarding independence of correlated hips/images across partitions
Klontzas et al. (2023) [30]	L	L	L	U	U	Independent external-country validation and expert consensus reference standard, but relatively limited external sample size
Li et al. (2023) [31]	U	L	L	U	U	One MRI slice per patient and training-only augmentation reduce leakage concerns; external validation was not reported
Ruckli et al. (2023) [32]	U	L	U	H	H	Very small single-center sample, no external validation, and patient-level grouping of multiple hips was not clearly reported
Shen et al. (2023) [33]	U	L	L	H	H	Large number of MRI segments derived from substantially fewer patients; patient-level separation across partitions was not explicitly reported
Shen et al. (2023) [34]	L	L	L	U	U	Large multicenter dataset with internal and external testing, but incomplete reporting of independence across patient/hip/segment levels
Shen et al. (2024) [35]	L	L	L	U	U	Multicenter, multivendor dataset with separate external institutional testing; some analytical/reporting uncertainties remain
Uemura et al. (2025) [36]	U	L	U	H	H	Small dataset, no external validation, principal ground-truth segmentation by one surgeon, and unclear patient-level grouping of bilateral hips
Wang et al. (2021) [37]	U	L	L	U	U	Patient-level train/test separation was reported, but validation remained internal and external generalizability was not assessed
Yan Liu et al. (2021) [38]	U	U	U	H	H	Limited reporting of dataset partitioning and methodological details, including handling of bilateral observations

## Data Availability

No new data were created or analyzed in this study. Data sharing is not applicable to this article.

## Supplementary Materials

The following supporting information can be downloaded at: https://www.mdpi.com/article/10.3390/bioengineering13080942/s1, Table S1: Complete database-specific search strategies.
