# Supplementary material for: Artificial Intelligence Applications in MRI for the Diagnosis and Management of Osteonecrosis of the Femoral Head: A Comprehensive Review
Source: Bioengineering (Basel). 2026 Aug 20;13(8):942. doi: 10.3390/bioengineering13080942 (PMC13509493; doi:10.3390/bioengineering13080942)
Supplement: Supplementary file 1 [file bioengineering-13-00942-s001.zip › bioengineering-4467869-supplementary.pdf]

bioengineering

MDPI

Supplementary Table S1. Complete database-specific search strategies

1. Database	2. Proposed complete search strategy
**PubMed/MEDLINE**	(("Osteonecrosis" OR "osteonecrosis of the femoral head" OR ONFH OR "avascular necrosis" OR "femoral head necrosis" AND ("Artificial Intelligence" OR "artificial intelligence" OR "machine learning" OR "deep learning" OR "convolutional neural network*" OR CNN) AND ("Magnetic Resonance Imaging" OR "magnetic resonance imaging" OR MRI))
**Scopus**	TITLE-ABS-KEY(("osteonecrosis of the femoral head" OR ONFH OR "avascular necrosis" OR "femoral head necrosis") AND ("artificial intelligence" OR "machine learning" OR "deep learning" OR "convolutional neural network" OR CNN) AND ("magnetic resonance imaging" OR MRI))
**Cochrane CENTRAL**	("osteonecrosis of the femoral head" OR ONFH OR "avascular necrosis" OR "femoral head necrosis") AND ("artificial intelligence" OR "machine learning" OR "deep learning" OR "convolutional neural network*" OR CNN) AND ("magnetic resonance imaging" OR MRI)
